# *In situ* X-ray absorption spectroscopy data during formation of active Pt- and Pd-sites in functionalized UiO-67 metal-organic frameworks

**DOI:** 10.1016/j.dib.2019.104280

**Published:** 2019-07-17

**Authors:** Aram L. Bugaev, Alina A. Skorynina, Elizaveta G. Kamyshova, Kirill A. Lomachenko, Alexander A. Guda, Alexander V. Soldatov, Carlo Lamberti

**Affiliations:** a The Smart Materials Research Institute, Southern Federal University, Sladkova 178/24, 344090, Rostov-on-Don, Russia; b European Synchrotron Radiation Facility, 71 Avenue des Martyrs, CS 40220, 38043 Grenoble Cedex 9, France; c Department of Physics and CrisDi Interdepartmental Centre, University of Turin, Via P. Giuria 1, 10125 Turin, Italy

**Keywords:** EXAFS, XANES, MOFs, Nanoparticles

## Abstract

We report a series of Pd *K*-edge and Pt *L*_3_-edge X-ray absorption spectra (XAS) collected in situ during thermal treatment of functionalized UiO-67-Pd and UiO-67-Pt metal-organic frameworks in inert and reducing atmospheres. We present raw synchrotron data from three subsequent experiments at different beamlines, normalized XAS spectra and *k*^2^-weighted oscillatory χ(*k*) functions extracted from one of the datasets. Pd *K*-edge spectra were collected for the samples in 5% H_2_/He, 3% H_2_/He and pure He in the temperature range from room temperature (RT) to 450 °C. Pt *L*_3_-edge were collected for the samples in 3% H_2_/He, 10% H_2_/He and pure He in the temperature range from RT to 300 °C. All spectra are reported together with the used atmosphere and temperature. For the analysis of all reported datasets, please see “Evolution of Pt and Pd species in functionalized UiO-67 metal-organic frameworks”. Fourier-analysis of Pd *K*-edge is reported in “Formation and growth of Pd nanoparticles in UiO-67 MOF by in situ EXAFS”.

**Table:** Specifications table

Subject area	*Physics, chemistry*
More specific subject area	*In situ spectroscopy of functionalized metal-organic frameworks*
Type of data	*Table, text file, figure*
How data was acquired	*X-ray absorption spectra were collected in transmission mode at BM01B, BM31 and BM23 beamlines of ESRF synchrotron.*
Data format	*Raw*
Experimental factors	*X-ray absorption spectra in transmission mode*
Experimental features	*UiO-67 metal organic frameworks functionalized by Pt and Pd activated in inert and reducing atmospheres*
Data source location	*Grenoble, France (45.209749, 5.688410)*
Data accessibility	*Data is provided with this article*
Related research article	*A. L. Bugaev, A. A. Skorynina, L. Braglia, K. A. Lomachenko, A. A. Guda, A. Lazzarini, S. Bordiga, U. Olsbye, K. P. Lillerud, A. V. Soldatov, C. Lamberti. Evolution of Pt and Pd species in functionalized UiO-67 metal-organic frameworks.* *Catalysis Today* https://doi.org/10.1016/j.cattod.2019.03.054

**Table:** 

**Value of the data** • Extensive datasets of X-ray absorption spectra collected at Pd *K*- and Pt L_3_-edges under various external conditions for functionalized UiO-67 metal-organic frameworks. • EXAFS data can be used for single- and multiple-shell Fourier analysis. • XANES data can be used to characterize the local atomic and electronic state of Pd and Pt atoms in the as synthesized functionalized UiO-67 and during treatment in inert and reducing atmospheres. • XANES spectra of the initial structures and intermediates can be utilized for databases, e.g. for implementation of machine learning approaches.

## 1 Data

**Fig. 1:**
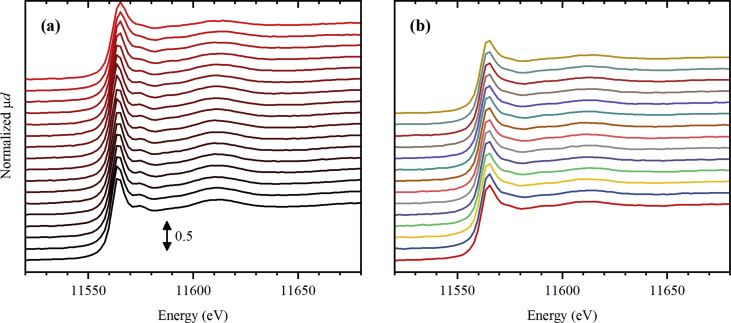
Normalized Pt *L*_3_-edge X-ray absorption near-edge structure (XANES) spectra collected for UiO-67 functionalized by Pt during activation in He (part a) from RT (black) to 300 °C (red), and subsequently collected (4 minutes per spectrum) at 300 °C (part b, from bottom to top).

**Fig. 2:**
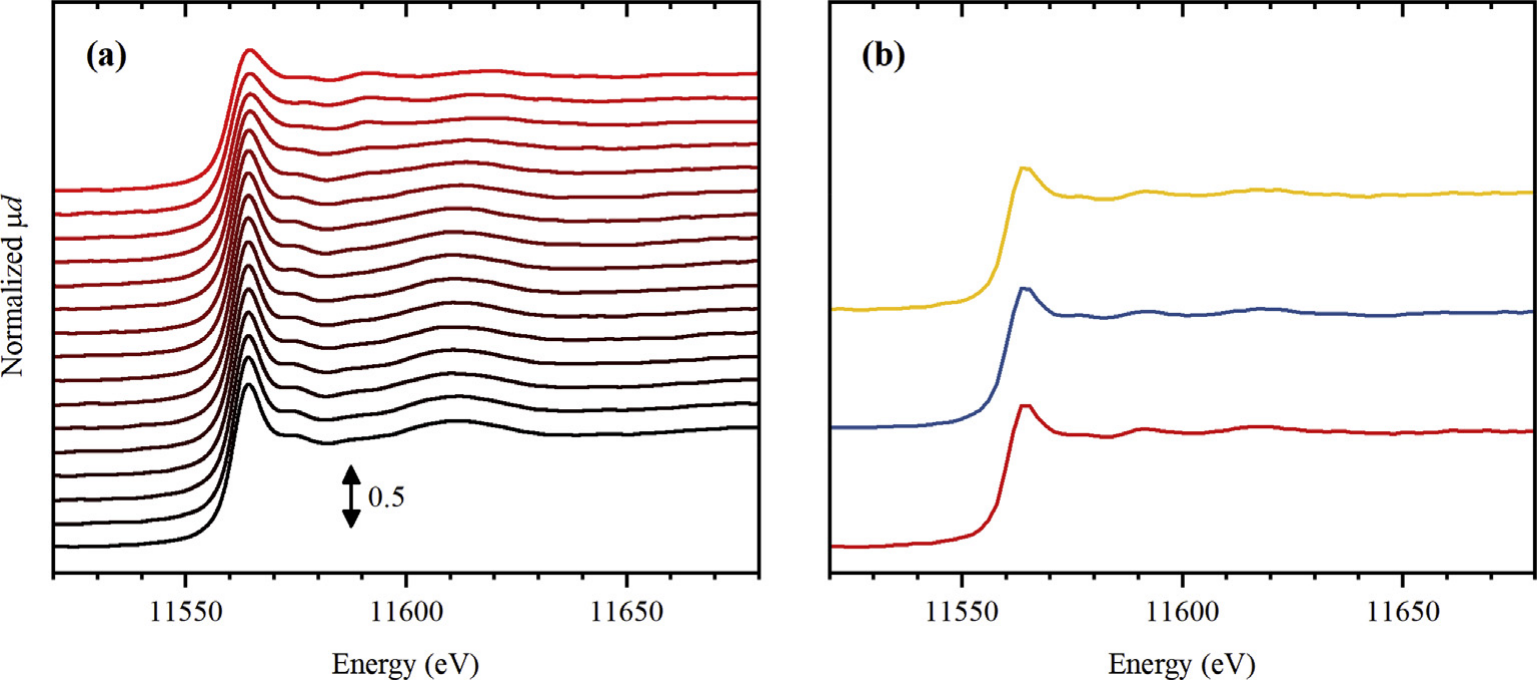
Normalized Pt *L*_3_-edge XANES spectra collected for UiO-67 functionalized by Pt during activation in 3% H_2_/He (part a) from RT (black) to 300 °C (red), and subsequently collected (4 minutes per spectrum) at 300 °C (part b, from bottom to top).

**Fig. 3:**
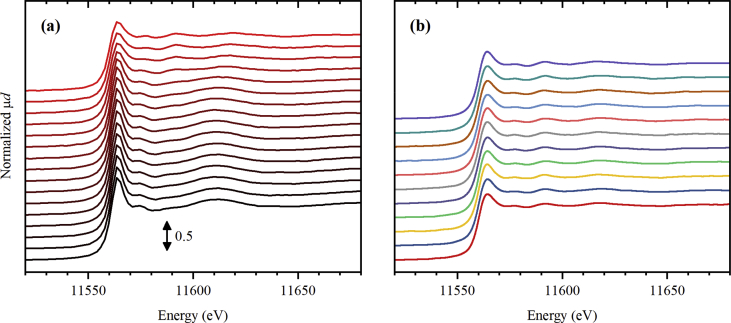
Normalized Pt *L*_3_-edge XANES spectra collected for UiO-67 functionalized by Pt during activation in 10% H_2_/He (part a) from RT (black) to 300 °C (red), and subsequently collected (4 minutes per spectrum) at 300 °C (part b, from bottom to top).

**Fig. 4:**
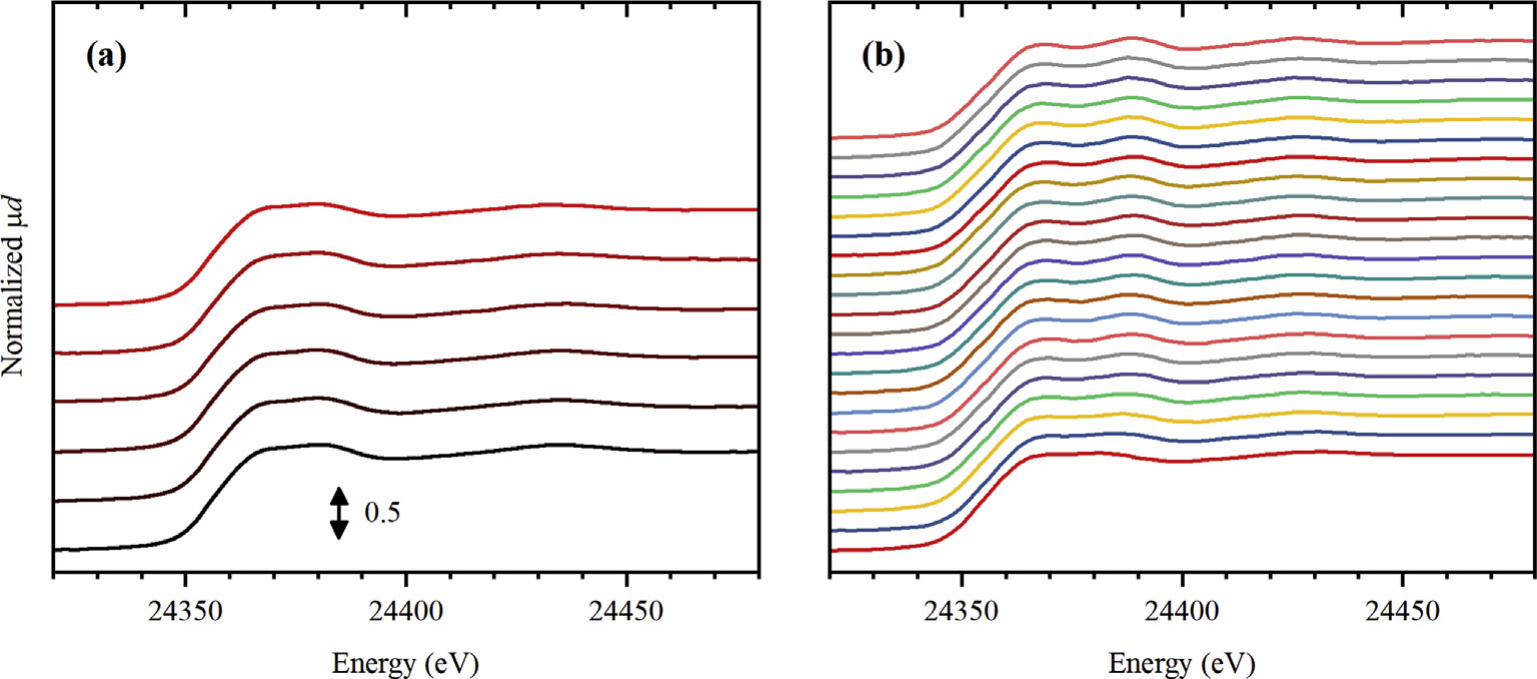
Normalized Pd *K*-edge XANES spectra collected for UiO-67 functionalized by Pd during activation in He (part a) from RT (black) to 300 °C (red), and subsequently collected (11 minutes per spectrum) at 300 °C (part b, from bottom to top).

**Fig. 5:**
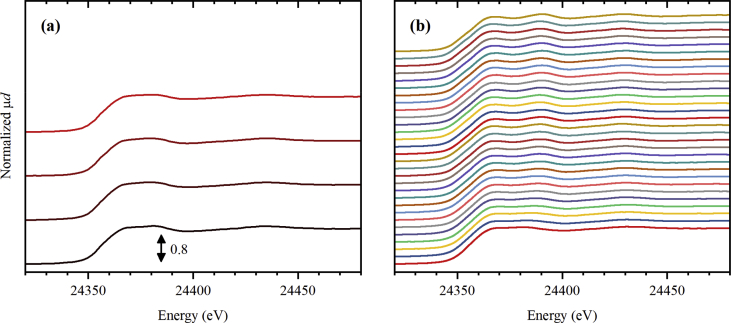
Normalized Pd *K*-edge XANES spectra collected for UiO-67 functionalized by Pd during activation in 3% H_2_/He (part a) from RT (black) to 215 °C (red), and subsequently collected (11 minutes per spectrum) at 215 °C (part b, from bottom to top).

**Fig. 6:**
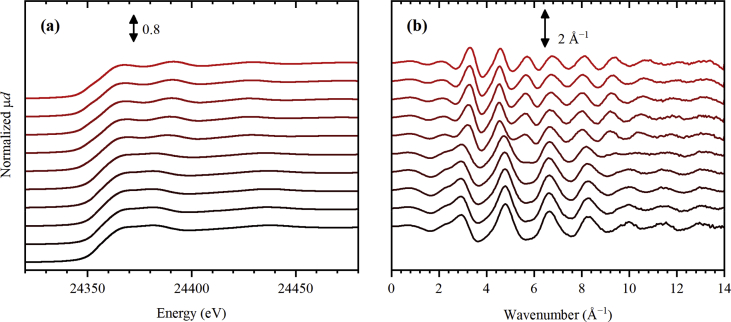
Normalized Pd *K*-edge XANES spectra (part a) and corresponding *k*^2^-weighted oscillatory χ(*k*) functions (part a) collected for UiO-67 functionalized by Pd in the pelletized form during activation in 5% H_2_/He from RT (black) to 450 °C (red).

The dataset contains 77 Pt *L*_3_-edge XAS spectra and 72 Pd *K*-edge XAS spectra collected during thermal treatment of UiO-67 samples functionalized by Pt [1] and Pd [1], [2], respectively, in inert (He) and reducing (H_2_/He) atmospheres with different H_2_/He content. The raw data is presented in the form of unnormalized X-ray absorption coefficient μ(*E*) (see files with extension *.mu in the Supporting materials) together with the normalized ones (see Fig. 1, Fig. 2, Fig. 3, Fig. 4, Fig. 5, Fig. 6 and files with extension *.norm in the Supporting materials). For 10 extended X-ray absorption fine structure (EXAFS) spectra collected at Pd *K*-edge, extracted *k*^2^-weighted oscillatory χ(*k*) functions are also reported in Fig. 6b and file “11_Pd_5H2_ramp.chik2” of the Supporting materials. The experimental conditions under which the spectra were collected are reported in Table 1, Table 2 for Pt *L*_3_-edge and, respectively, Pd *K*-edge data.

**Table 1:** Experimental conditions applied during Pt *L*_3_-edge spectra collection and their correspondence with the column in *.mu and *.norm files. *T* is the temperature in °C registered in the beginning of each spectrum acquired during the ramp. *t* is the time in minutes, the sample had spent at 300 °C before the beginning of the corresponding spectrum. The first and second columns in each file correspond to energy in eV and absorption spectrum of the reference Pt foil, respectively.

Conditions Column	He		3% H_2_/He		10% H_2_/He	
	*T*, °C	*t*, min	*T*, °C	*t*, min	*T*, °C	*t*, min
3	20	0	22	0	22	0
4	22	4	40	4	40	4
5	40	8	58	8	58	8
6	58	12	77		77	12
7	77	16	95		95	16
8	95	20	113		113	20
9	113	24	131		131	37
10	131	28	149		149	41
11	149	32	167		167	45
12	167	36	185		185	49
13	185	40	203		203	53
14	203	44	221		221	
15	221	48	239		239	
16	239	52	257		257	
17	257		274		274	
18	274		293		293	
19	293

**Table 2:** Experimental conditions applied during Pd *K*-edge spectra collection and their correspondence with the column in *.mu and *.norm files. *T* is the temperature at which the spectra were °C registered. *t* is the time in minutes, the sample had spent at 300 and 215 °C in He and 3% H_2_/He, respectively, before the beginning of the corresponding spectrum. The first and second columns in each file correspond to energy in eV and absorption spectrum of the reference Pd foil, respectively.

Conditions column	He		3% H_2_/He	
	*T*, °C	*t*, min	*T*, °C	*t*, min
3	46	0	46	0
4	89	11	89	11
5	131	22	131	22
6	174	33	174	33
7	217	44		44
8	260	55		55
9		66		69
10		77		80
11		88		91
12		99		102
13		110		133
14		121		144
15		132		155
16		143		166
17		154		177
18		165		188
19		176		199
20		187		210
21		198		221
22		209		232
23		220		250
24		231		261
25				272
26				283
27				294
28				305
29				316
30				327
31				338
32				349

## 2 Experimental design, materials, and methods

**Samples.** The sample are metal-organic frameworks of UiO-67 type. by palladium and platinum was achieved via substitution of 10% standard bpdc linkers by MCl_2_bpydc (M = Pd, Pt) ones using the pre-made linker synthesis (PMLS) approach [3]. The synthesis procedure have been described in more detail in our previous works for both Pd [4] and Pt [5], [6].

**Pt *L***_**3**_**-edge XAS spectra** for UiO-67-Pt samples (Fig. 1, Fig. 2, Fig. 3) were collected at BM01B beamline [7] (now moved to BM31 port) of ESRF. The sample powder was loaded inside a 1.5 mm capillary and fixed by the quartz wool from both sizes. The mass of the samples was varied from 2.4 to 2.8 mg in the three subsequent experiments with different treatment procedures. The capillary was glued inside a metal holder, which was then connected to a remotely controlled gas line, equipped with Bronkhorst mass flow controllers. Below the sample, there was a gas blower mounted, calibrated using a thermocouple. The sample was heated from room temperature (RT) to 300 °C with the ramp of 5 °C/min. The total flux of the gas through the capillary was adjusted to 1.4 mL/min, which was checked by the mass flow meter. Three different gas mixtures were sent: pure He (Figs. 1), 3% H_2_/He (Figs. 2), and 10% H_2_/He (Fig. 3). XAS spectra were collected continuously during the ramp and after reaching 300 °C. The photon energy was scanned from 11.35 to 12.42 keV by Si(111) double crystal monochromator operated in continuous scanning mode. In such mode, one full spectrum was collected in 4 minutes. The rejection of higher harmonics was achieved by detuning of the second crystal until 60% of the maximal intensity (when both crystals are perfectly tuned) was observed.

**Pd *K*-edge XAS spectra** for UiO-67-Pd samples (Fig. 4, Fig. 5) were collected at BM31 beamline [7] ESRF, using a similar setup as described above for Pt *L*_3_-edge. The mass of the sample inside the capillaries was around 5 mg. The samples were sieved before loading into the capillaries and the fraction below 100 μm was removed. The total flux of 50 mL/min was applied. Two different gas mixtures were sent: pure He (Figs. 4), 3% H_2_/He (Fig. 5). The samples were first heated stepwise until no spectral changes were observed and were then kept at 300 and 215 °C in inert and reducing flux, respectively, and the spectra were measured continuously. The photon energy was scanned from 24.0 to 25.4 keV using Si(111) double crystal monochromator operated in continuous scanning mode. In such mode, one full spectrum was collected in 11 minutes. The rejection of higher harmonics was achieved by detuning of the second crystal until 80% of the maximal intensity (when both crystals are perfectly tuned) was observed.

Additional measurements were performed at BM23 beamline of ESRF using the sample in a pelletized form to optimize the absorption step and collect also high-quality EXAFS data (Fig. 6). A pelletized sample was held in a microtomo cell [8] and was activated in a flow of 5% H_2_/He (50 mL/min). A double-crystal fixed-exit Si(111) monochromator was employed. Harmonic rejection was done by two flat Pt-coated mirrors positioned at 2 mrad angle. The spectra were collected in the energy range from 24.1 to 25.1 eV, which correspond to *k*_max_ of about 14 Å^−1^. The energy step in the pre-edge region was set to 5 eV with acquisition time of 1 s per point. In XANES region, 1 eV step was used with 1 s/point. In the EXAFS region, the step of 0.04 Å^−1^ in the *k*-space was used, with the time per point increasing linearly from 1 to 4 s.

All spectra were collected in transmission mode, and Pt and Pd foils were measured simultaneously with third ionization chamber for energy calibration. Demeter software [9] was used to normalize the data and to obtain oscillatory χ(*k*) functions reported in Fig. 6a. The capillary setups used at BM01B and BM31 also allowed quasi-simultaneous collection of X-ray diffraction as described elsewhere [10], [11], [12], [13].
